# Efficacy and safety analysis of montelukast sodium added on azithromycin in the treatment of *Mycoplasma pneumoniae* pneumonia in children

**DOI:** 10.3389/fmed.2025.1506621

**Published:** 2025-06-27

**Authors:** Ying Liang, Wei Li

**Affiliations:** ^1^Department of Pediatric Medicine, Shanghai Sixth People's Hospital, Shanghai Jiao Tong University School of Medicine, Shanghai, China; ^2^Department of Pediatric Medicine, Shanghai General Hospital, Shanghai Jiao Tong University School of Medicine, Shanghai, China

**Keywords:** *Mycoplasma pneumoniae* pneumonia, azithromycin, montelukast sodium, therapeutic effect, security

## Abstract

**Objective:**

To evaluate the safety and effectiveness of using montelukast sodium added on azithromycin for the treatment of pediatric *Mycoplasma pneumoniae* pneumonia (MPP).

**Methods:**

Retrospective analysis showed that 122 children with MPP were divided into a control group (61 cases received azithromycin treatment) and a study group (61 cases received azithromycin combined with montelukast sodium treatment) according to different treatment plans. Both groups were treated for 7 days, and lung function, lung compliance, chest CT signal, inflammatory immune status, and side effects were compared before and after treatment.

**Result:**

After a month of treatment, positive end expiratory pressure (PEEP) levels were lower in the research group compared to the control group’s kids, whereas forced vital capacity (FVC), tidal volume (TV), peak expiratory flow rate (PEF), pulmonary ventilation per minute (MVV), and pulmonary dynamic compliance (Cdyn) levels were greater (*p* < 0.05). After a month of treatment, the detection rates of lung CT signs such as bronchial wall thickening, pleural effusion (PE), ground glass density shadow (GGO), gravel sign, lymphadenopathy, consolidation of lung (LC) and bronchogenic sign in the research group were all lower than those in the control group (*p* < 0.05). Compared to the control group, serum levels of amyloid A (SAA), procalcitonin (PCT), lactate dehydrogenase (LDH), TOll-like receptor 4 (TLR4), and CD8 + were all lower in the research group.

**Conclusion:**

Azithromycin plus montelukast sodium can effectively improve the lung function and lung compliance of children with MPP, reduce the detection rate of chest CT signs of disease symptoms, and improve the inflammatory and immune state of the body, without increasing adverse reactions, and the clinical application is safe.

## 1 Introduction

*Mycoplasma pneumoniae* pneumonia (MPP) is a disease that occurs mostly in school-age children. Some factors (such as poor air flow, low immunity, etc.) induce the infection of *Mycoplasma pneumoniae* (MP), which then causes invasion and injury to respiratory tract and lung tissue. Children often show severe dry cough, fever and sore throat (1, 2). Macrolide antibiotics (MA), such as azithromycin, were mostly used in the clinical treatment of MPP in the past, which can reduce the activity of mycoplasma and the overall number by inhibiting the synthesis process of bacterial protein, thus alleviating the clinical symptoms of MPP (3, 4). However, it has been reported that in recent years, with the wide use of azithromycin in the treatment process, macrolide-resistant (MR) strains began to appear and the number showed an increasing trend. Some MPP children were resistant to azithromycin alone and could not achieve the expected clinical efficacy (5). Therefore, combined with clinical practice, it is necessary to consider the combined medication scheme. Montelukast sodium is a new non-hormonal anti-inflammatory drug, which belongs to leukotriene receptor antagonist (LTRA). It can specifically bind with cysteinyl leukotriene (CysLTs), an important inflammatory mediator receptor, to reduce the body’s inflammatory response and tracheal mucosal injury, thus reducing airway hyperresponsiveness (AHR) and promoting the relief or disappearance of symptoms such as wheezing and cough in children (6, 7). In view of this, this study applied azithromycin combined with montelukast sodium in the treatment of MPP in children, and observed its effects on lung compliance, chest CT findings, inflammatory immune status and so on. Shown as follows.

## 2 Data and methods

### 2.1 General information

After a retrospective analysis, the clinical data of 122 children with MPP who received hospital treatment between June 2022 and June 2024 were split into two groups based on various treatment plans: the research group (61 cases treated with Azithromycin combined with Montelukast sodium for treatment sodium) and control group (61 cases treated with azithromycin). The sex, age, body weight, heart rate (HR), height, respiratory rate (RR), alanine aminotransferase (ALT), creatinine (Scr), uric acid (UA) and the time from onset to treatment between the two groups were well balanced (*p* > 0.05). See Table 1.

**Table 1:** Comparison of two groups of general data.

Index		Research group (*n* = 61)	Control group (*n* = 61)	Statistical values	*p*
Gender [*n*(%)]	Man	33(54.10)	35(57.38)	*χ*^2^ = 0.133	0.715
	Woman	28(45.90)	26(42.62)		
Age ( $\bar{x}$ ± *s*, years old)		6.71 ± 1.02	6.74 ± 1.03	*t* = 0.162	0.872
Body mass ( $\bar{x}$ ± *s*, kg)		22.18 ± 1.49	22.25 ± 1.52	*t* = 0.257	0.798
HR at admission ( $\bar{x}$ ±*s*, times /min)		89.63 ± 6.27	90.05 ± 6.34	*t* = 0.368	0.714
height ( $\bar{x}$ ± *s*, cm)		121.63 ± 5.41	122.04 ± 5.35	*t* = 0.421	0.675
RR at admission ( $\bar{x}$ ± *s*, times /min)		20.28 ± 1.16	20.37 ± 1.18	*t* = 0.425	0.672
ALT at admission ( $\bar{x}$ ± *s*, U/L)		16.12 ± 0.53	16.25 ± 0.51	*t* = 1.380	0.170
Scr at admission ( $\bar{x}$ ± *s*, μmol/L)		28.52 ± 2.61	28.71 ± 2.54	*t* = 0.408	0.684
UA at admission ( $\bar{x}$ ± *s*, μmol/L)		283.34 ± 19.55	284.06 ± 20.08	*t* = 0.201	0.841
Time from onset to treatment ( $\bar{x}$ ± *s*, d)		2.13 ± 0.41	2.22 ± 0.43	*t* = 1.183	0.239

HR: heart rate; RR: respiratory rate; ALT: alanine transaminase; SCr: serum creatinine; UA: uric acid.

### 2.2 Entry criteria

(1) Inclusion criteria: meeting MPP diagnostic criteria (8) and being positive for MP antigen by throat swab; According to the Diagnosis and Treatment Guidelines for Mycoplasma Pneumonia in Children (2023 Edition), it is classified as mild MPP; All patients were accompanied by different degrees of fever, wheezing, cough and other clinical symptoms; The clinical data of the children are complete; The time from onset to treatment is less than <7d; days; 5 years old < children’s age < 12 years old; After the onset, he was admitted to the hospital for the first time and did not take medication privately.(2) Exclusion criteria: the compliance of children with treatment and examination is poor or there are contraindications for imaging examination; Combined with congenital immunodeficiency, congenital bronchial malformation, liver and kidney dysfunction or chronic respiratory diseases; Complicated with other pathogen infection, serious infection in other parts or other respiratory related diseases; Allergic to azithromycin or montelukast sodium; The condition belongs to difficult to treat MPP and severe MPP in children; Complicated with tuberculosis and congenital heart disease; Cough and asthma symptoms caused by other factors (such as allergic diseases, bronchial foreign bodies, etc.); Combined with circulatory failure or respiratory failure.

### 2.3 Methods

After admission, the children in the two groups were given routine basic symptomatic treatment and nursing according to their specific physical conditions, including relieving cough and phlegm, relieving spasm and asthma, reducing temperature and fever, intermittent oxygen inhalation, correcting water and electrolyte disorder, correcting acid–base balance, and improving related laboratory or imaging examination.

#### 2.3.1 Control group

The control group received azithromycin injection (Shenzhen Haiwang Pharmaceutical Co., Ltd., National Medical Products Administration Standard H20010701, specification: 2 mL: 0.1 g) is added to 250 mL or 500 mL of 0.9% sodium chloride injection or 5% glucose injection to achieve a final concentration of azithromycin of 1.0–2.0 mg/mL. It is administered intravenously for at least 60 min. The treatment for pediatric patients is 10 mg/kg/day, once daily for 3 consecutive days, and may be extended to 5 days if necessary, once daily.

#### 2.3.2 Research group

The research group implemented a combination regimen of Montelukast Sodium and Azithromycin. The dosage and administration of Azithromycin Injection were the same as those of the control group. Montelukast Sodium Chewable Tablets (Unacon Ouyi Pharmaceutical Co., Ltd., specification: 5 mg/tablet, national drug standard H20203048) were used. The dosage was calculated based on the age of the patient (9), with the children aged ≤6 years old being 4 mg/ time and the children aged > 6 years old being 5 mg/ time, once a day, The medication course is 7 days.

### 2.4 Observation indicators

(1) Lung function and dynamic lung compliance (Cdyn). Lung function detector (Schiller Medical Equipment, Switzerland, PowerCube-Body model, National Machinery Injection 20,162,072,421) was used to monitor and calculate the positive end expiratory pressure (PEEP), forced vital capacity (FVC), tidal volume (TV), peak expiratory flow rate (PEF), minute lung ventilation (MVV) and Cdyn level of the two groups. Each index was tested three times, and the results were taken as the average value of three times. Where Cdyn = TV/ (platform pressure -PEEP). (2) The detection rate of chest CT signs. For a month, the two groups’ rates of chest CT sign detection were compared before and after treatment. The child was taken in supine position, and the whole lung field of the child was scanned with a CT machine (SOMATOM Go, 64 rows, 128 floors, Siemens, Germany), and the internal structure, shape and distribution of the lesion were observed, and the main CT signs of the diseased lung were recorded, including bronchial wall thickening, pleural effusion (PE), ground glass density shadow (GGO) in the lung, gravel sign and so on. The imaging data of the above cases were jointly reviewed by two senior radiologists using a double-blind method. (3) Inflammatory immune state. Before and 7 days after therapy, 5 mL of fasting venous blood were collected. Using a centrifuge (Jinan Tianqinhao Biotechnology, TG-21WC) with a radius of 10 cm and a speed of 3,000 rpm for 10 min, routine anticoagulation was carried out. The upper serum was then separated and kept for testing in a low-temperature refrigerator. Serum levels of amyloid A (SAA), procalcitonin (PCT), lactate dehydrogenase (LDH) and TOll-like receptor 4(TLR4) were detected by ELISA (SAA kit was purchased from Guangzhou Orida Biotechnology, PCT kit was purchased from Shanghai Copery Biotechnology, LDH kit was purchased from Tianjin Peptide Chain Biotechnology, and TLR4 kit was purchased from Shanghai Yubo Biotechnology). The levels of CD4 + and CD8 + in serum of two groups were detected by flow cytometry (Partec Medical, CyFlow Cube8). (4) Adverse reactions. Throughout the two groups’ treatments, keep an eye out for the Diarrhea and abdominal pain, Nausea and vomiting, Itch, Dizzy, Rash, Hypersomnia.

### 2.5 Statistical methods

Using SPSS23.0 software, the measurement data are expressed by ( $\bar{x}$ ± *s*) and tested by *t*; The counting data was represented by *n*(%), and the difference was statistically significant with *χ*^2^ test, *p* < 0.05.

## 3 Results

### 3.1 Comparison of pulmonary function and Cdyn between two groups

**Figure 1:**
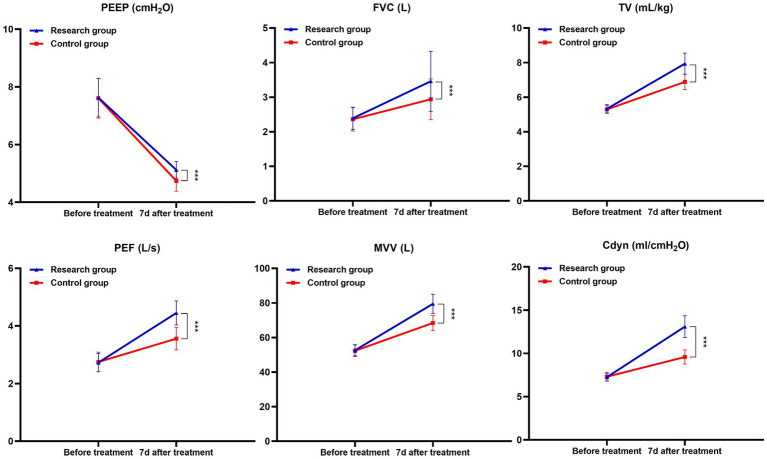
Changes in lung function and Cdyn before and after treatment in both groups (^***^p < 0.001).

After a month of treatment, the levels of FVC, TV, PEF, MVV and Cdyn in both groups of patients all increased compared with those before treatment, while the PEEP level decreased. Moreover, the increase in FVC, TV, PEF, MVV and Cdyn and the decrease in PEEP in the research group after a month of treatment were all greater than those in the control group (*p* < 0.05). See Table 2 and Figure 1.

**Table 2:** Comparison of pulmonary function and Cdyn between the two groups ( $\bar{x}$ ± *s*).

Group	PEEP(cmH_2_O)		FVC(L)		TV(mL/kg)	
	Before treatment	After a month of treatment	Before treatment	After a month of treatment	Before treatment	After a month of treatment
Research group (*n* = 61)	7.63 ± 0.67	4.74 ± 0.36^*^	2.39 ± 0.32	3.46 ± 0.87^*^	5.33 ± 0.24	7.94 ± 0.61^*^
Control group (*n* = 61)	7.61 ± 0.69	5.12 ± 0.29^*^	2.36 ± 0.34	2.94 ± 0.59^*^	5.29 ± 0.23	6.88 ± 0.45^*^
*t*	0.162	6.420	0.502	3.864	0.940	10.922
*P*	0.871	0.000	0.617	0.000	0.349	0.000

Group	PEF(L/s)		MVV(L)		Cdyn(ml/cmH_2_O)	
	Before treatment	After a month of treatment	Before treatment	After a month of treatment	Before treatment	After a month of treatment
Research group (*n* = 61)	2.73 ± 0.32	4.45 ± 0.42^*^	52.65 ± 3.24	79.48 ± 5.56^*^	7.26 ± 0.47	13.09 ± 1.26^*^
Control group (*n* = 61)	2.75 ± 0.34	3.56 ± 0.39^*^	52.33 ± 3.37	68.41 ± 4.29^*^	7.31 ± 0.48	9.58 ± 0.82^*^
*t*	0.335	12.128	0.535	12.312	0.581	18.236
*P*	0.739	0.000	0.594	0.000	0.562	0.000

Compared with before treatment, ^*^P < 0.05; PEEP: positive end expiratory pressure; FVC: forced vital capacity; TV: tidal volume; PEF: peak expiratory flow rate; MVV: minute lung ventilation; Cdyn: dynamic lung compliance.

### 3.2 Comparison of the detection rate of chest CT signs between the two groups

**Figure 2:**
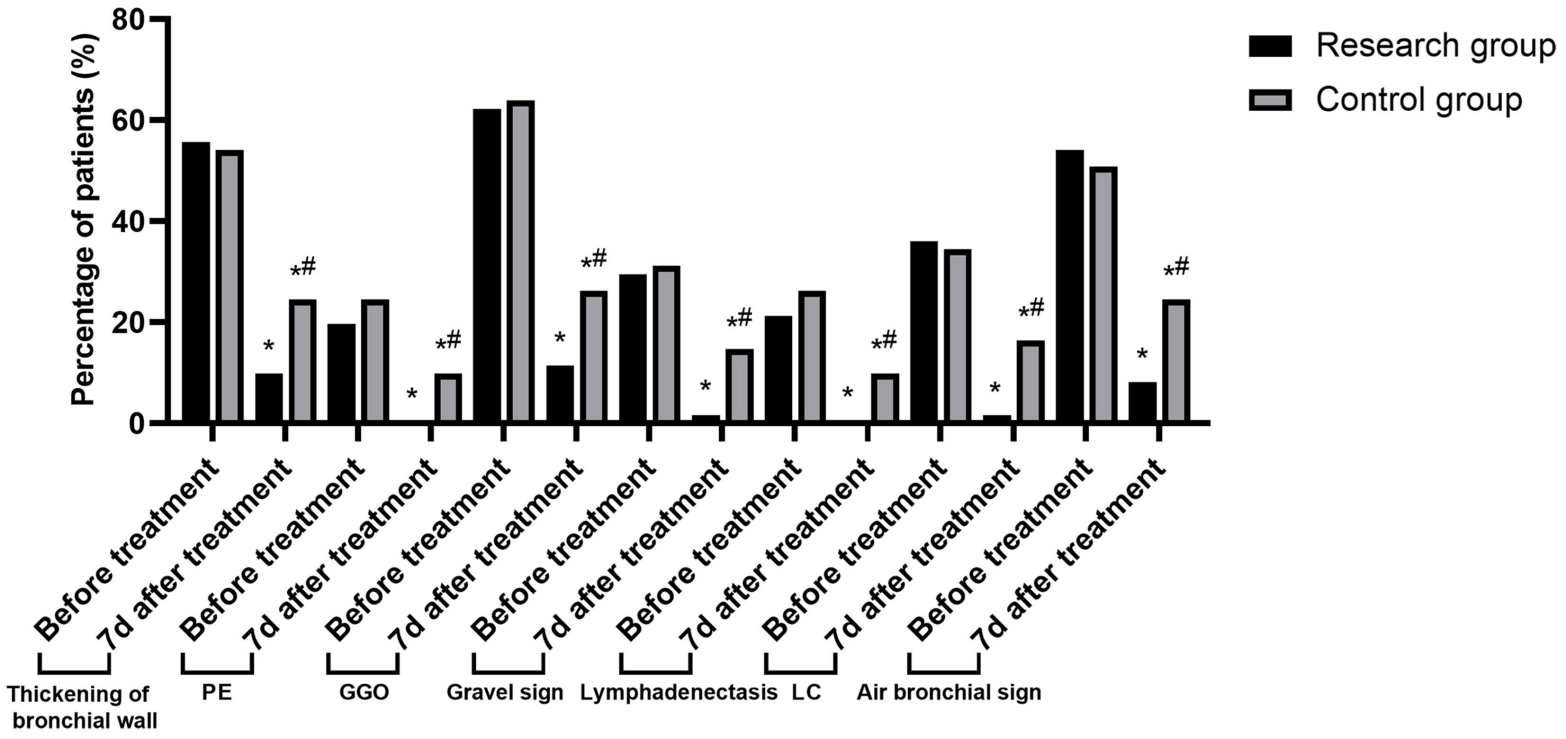
Changes in the detection rate of lung CT signs in the two groups before and after treatment (Compared to before treatment, ^*^p < 0.05; Compared to the research group, ^#^*p* < 0.05).

After a month of treatment, both groups’ rates of pulmonary CT sign detection—including bronchial wall thickening, PE, GGO, lithotripsy sign, lymphadenopathy, LC, and bronchogenic sign—were lower than pre-treatment levels. Moreover, children in the research group had lower detection rates than those in the control group (*p* < 0.05). See Table 3 and Figure 2.

**Table 3:** Comparison of the detection rate of pulmonary CT signs between the two groups before and after treatment for 7 days [n(%)].

Group	Thickening of bronchial wall		PE		GGO		Gravel sign	
	Before treatment	After a month of treatment	Before treatment	After a month of treatment	Before treatment	After a month of treatment	Before treatment	After a month of treatment
Research group (*n* = 61)	34(55.74)	6(9.84)^*^	12(19.67)	0(0.00)^*^	38(62.30)	7(11.48)^*^	18(29.51)	1(1.64)^*^
Control group (*n* = 61)	33(54.10)	15(24.59)^*^	15(24.59)	6(9.84)^*^	39(63.93)	16(26.23)^*^	19(31.15)	9(14.75)^*^
*χ* ^2^	0.033	4.659	0.428	4.382	0.035	4.340	0.039	6.971
*P*	0.856	0.031	0.513	0.036	0.851	0.037	0.844	0.008

Group	Lymphadenectasis		LC		Air bronchial sign	
	Before treatment	After a month of treatment	Before treatment	After a month of treatment	Before treatment	After a month of treatment
Research group (*n* = 61)	13(21.31)	0(0.00)^*^	22(36.07)	1(1.64)^*^	33(54.10)	5(8.20)^*^
Control group (*n* = 61)	16(26.23)	6(9.84)^*^	21(34.43)	10(16.39)^*^	31(50.82)	15(24.59)^*^
*χ* ^2^	0.407	4.382	0.036	8.093	0.132	5.980
*P*	0.523	0.036	0.850	0.004	0.717	0.015

Compared with before treatment, ^*^P < 0.05; PE: pleural effusion; GGO: ground glass density shadow; LC: consolidation of lung.

### 3.3 Comparison of inflammatory immune status between the two groups

**Figure 3:**
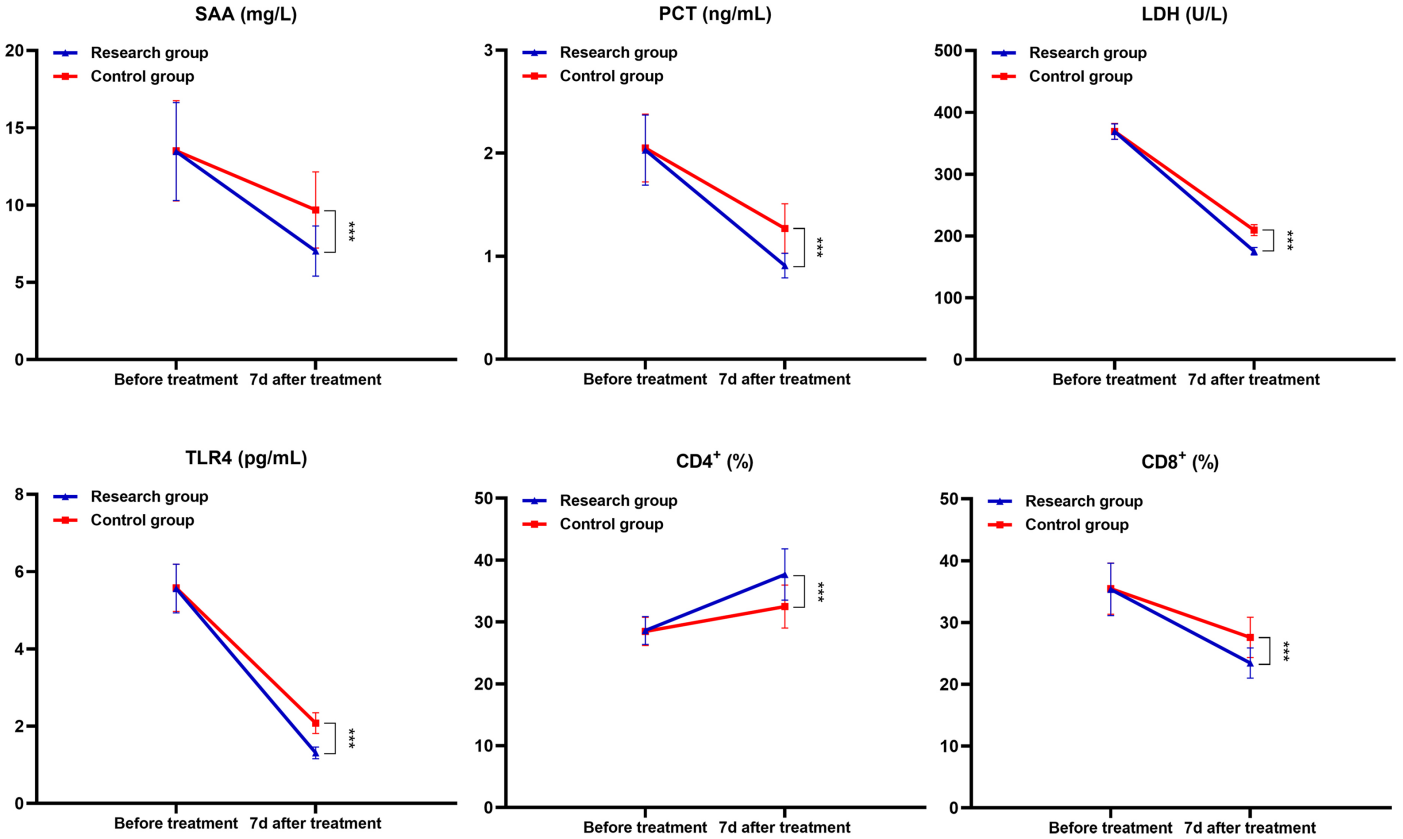
Comparison of inflammatory and immune status related indexes between the two groups (^***^p < 0.001).

After 7 days of therapy, the research group’s serum SAA, PCT, LDH, TLR4, and CD8+ levels were lower and CD4+ was greater (*p* < 0.05) than those of the children in the control group. See Table 4 and Figure 3.

**Table 4:** Comparison of inflammatory and immune status related indexes between the two groups ( $\bar{x}$ ± *s*).

Group	SAA(mg/L)		PCT(ng/mL)		LDH(U/L)	
	Before treatment	After 7 days of treatment	Before treatment	After 7 days of treatment	Before treatment	After 7 days of treatment
Research group (*n* = 61)	13.46 ± 3.17	7.03 ± 1.62^*^	2.03 ± 0.34	0.91 ± 0.12^*^	368.79 ± 12.57	175.31 ± 6.25^*^
Control group (*n* = 61)	13.51 ± 3.24	9.68 ± 2.47^*^	2.05 ± 0.33	1.27 ± 0.24^*^	369.12 ± 12.66	209.43 ± 8.79^*^
*t*	0.086	7.007	0.330	10.479	0.145	24.708
*P*	0.932	0.000	0.742	0.000	0.885	0.000

Group	TLR4(pg/mL)		CD4^+^(%)		CD8^+^(%)	
	Before treatment	After 7 days of treatment	Before treatment	After 7 days of treatment	Before treatment	After 7 days of treatment
Research group (*n* = 61)	5.56 ± 0.63	1.31 ± 0.15^*^	28.62 ± 2.27	37.67 ± 4.15^*^	35.36 ± 4.25	23.44 ± 2.45^*^
Control group (*n* = 61)	5.58 ± 0.61	2.08 ± 0.27^*^	28.46 ± 2.31	32.51 ± 3.48^*^	35.47 ± 4.14	27.59 ± 3.26^*^
*t*	0.178	19.471	0.386	7.441	0.145	7.948
*P*	0.859	0.000	0.700	0.000	0.885	0.000

Compared with before treatment, ^*^P < 0.05; SAA: Serum levels of amyloid A; PCT: procalcitonin; LDH: lactate dehydrogenase; TLR4: TOll-like receptor 4.

### 3.4 Comparison of adverse reactions between the two groups

The incidence of adverse responses did not significantly differ between the research group’s children (9.84%) and the control group’s children (4.92%) (*p* > 0.05). See Table 5.

**Table 5:** Comparison of adverse reactions between the two groups [*n*(%)].

Group	Diarrhea and abdominal pain	Nausea and vomiting	Itch	Dizzy	Rash	Hypersomnia	total
Research group (*n* = 61)	1(1.64)	0(0.00)	1(1.64)	0(0.00)	0(0.00)	1(1.64)	3(4.92)
Control group (*n* = 61)	1(1.64)	1(1.64)	1(1.64)	1(1.64)	1(1.64)	1(1.64)	6(9.84)
*χ* ^2^							0.480
*P*							0.489

## 4 Discussion

MPP has the characteristics of acute onset and rapid progress. If effective treatment measures are not taken in time, it may develop into refractory MPP or critical MPP with the progress of the disease, involving a large area of lung tissue and extrapulmonary organs, threatening the health and quality of life of children. At present, although there are many drug choices in clinical treatment of MPP, there is still a lack of unified treatment scheme in view of children’s special physical condition.

Related research shows that when MPP occurs, children’s lung function is affected to some extent and their lung compliance is reduced accordingly (10, 11). Chest CT examination can give full play to the advantages of high spatial and density resolution with the help of reconstruction technology, and clearly display the characteristics of lung lesions, pleura, mediastinum and so on, thus providing reliable basis for clinical diagnosis of MPP and differential diagnosis of other types of pneumonia (12). The results of this study show that after a month of treatment, compared with the control group, the level of PEEP in the study group is lower, the levels of FVC, TV, PEF, MVV and Cdyn are higher, and the detection rate of chest CT signs such as bronchial wall thickening, PE and GGO is less, suggesting that azithromycin + montelukast sodium can improve children’s lung function, improve lung compliance and reduce the detection rate of chest CT signs of MPP symptoms. The analysis reason is that azithromycin is a kind of MA drug, which has good tissue permeability. After intravenous administration, it can quickly exert its efficacy, and it closely binds with the 50s large subunit of MP, which hinders the synthesis of MP protein and helps to clear MP, thus effectively reducing or blocking the continuous secretion of inflammatory factors in the body caused by MP infection and alleviating the inflammatory reaction in the lungs (13). In addition, azithromycin has a long half-life, and it can continue to release its drug properties even after stopping taking the drug, maintaining a high concentration level of drugs in cells for a long time. In addition, the drug mainly acts on inflammatory lesions such as bronchi and lungs, and the targeted effect of eliminating germs is remarkable. As an LTRA drug, montelukast sodium is highly selective and competitive to CysLTs, which can block leukotrienes (LTs) from combining with CysLTs. LTs is a group of inflammatory mediators in the metabolism of arachidonic acid (AA), which can stimulate mucus secretion and airway smooth muscle contraction. MPP infection can induce airway hyperresponsiveness, resulting in cough, wheezing and other symptoms. Montelukast sodium can reduce airway smooth muscle spasm and airway resistance by inhibiting LTs, thus improving patients’ ventilation function and lung function. In addition, by inhibiting the infiltration of inflammatory cells such as neutrophils and eosinophils, montelukast sodium helps to reduce collagen deposition and delay the change of airway structure, so as to promote the absorption of inflammatory lesions such as ground glass shadow and improve lung imaging (14).

The occurrence and development of MPP is closely related to the pulmonary inflammatory reaction caused by MP infection. When MP infects the respiratory tract, it can inhibit the mucociliary clearance movement, cause the over-expression of cell adhesion factors in the respiratory tract, and cause abnormal immune response (15). SAA is an acute phase protein, PCT can reflect the active degree of systemic inflammatory response, and TLR4 is a transmembrane protein and an important immune system receptor, which can induce immune inflammatory response (16). The results of this study also showed that compared with the control group, the serum SAA, PCT, LDH, TLR4 and CD8 + levels of children in the study group were lower and CD4 + levels were higher after 7 days of treatment, suggesting that azithromycin + montelukast sodium can effectively improve the inflammatory immune status of children with MPP. The reason is that MP has the characteristics of no cell wall and abundant protein, and azithromycin has a strong inhibitory effect on its protein synthesis, which can lead to the wrong aggregation and folding of MP protein by affecting the transpeptidase in MP protein synthesis, thus alleviating lung inflammation. Although azithromycin has remarkable efficacy, it has no decomposition effect on secretions blocked in bronchi, so combining with other drugs is beneficial to comprehensive drug advantages and improve clinical treatment effect. The competitive combination of montelukast sodium and CysLTs can reduce the activity of LTs polypeptide, thus reducing or blocking the release and infiltration of various inflammatory factors, reducing capillary circulation, effectively relieving airway inflammation, and promoting the improvement of immune function in children (17, 18).

There was no significant difference in adverse reactions between the two groups, which confirmed that azithromycin + montelukast sodium was safe in the treatment of MPP in children. The reason may be that azithromycin has the characteristics of stable chemical structure, high bioavailability and long half-life, and its half-life can usually reach 35-48 h, so it only needs to be taken once a day, and most intravenous drugs can be excreted in urine, so the risk of adverse reactions is low (19, 20). The average half-life of Montelukast sodium chewable tablets is 2.7–5.5 h. After entering the human body, it generally has good tolerance and mild adverse reactions, the overall incidence of adverse reactions is similar to that of placebo, ensuring the safety of treatment.

This study has certain limitations: First, this is a retrospective study that relies on historical medical records to extract data. There may be issues such as inconsistent record formats and missing key information, which could lead to biases in the assessment of intervention doses. Further prospective randomized controlled trials are still needed. Second, the sample size of this study is relatively small, which may result in the research conclusions not being universally applicable. It is still necessary to further increase the sample size. Thirdly, CT only evaluated the presence of pleural effusion and ground-glass opacity, without data on the severity of these pathologies, which might affect the research results. Fourth, in this study, the CT examination was not an enhanced CT and no contrast agent was used. This is not the best choice for detecting lymph node enlargement and may have an impact on the examination results of lymph node enlargement.

## 5 Conclusion

In conclusion, the application of azithromycin and montelukast sodium in the treatment of MPP in children is beneficial for improving the lung function, lung compliance, and the inflammatory and immune status of the body in children, and is conducive to reducing the detection rate of MPP symptoms on chest CT. Moreover, it does not increase the risk of adverse reactions.

## Data Availability

The raw data supporting the conclusions of this article will be made available by the authors, without undue reservation.
